# X-ray detectors at the Linac Coherent Light Source

**DOI:** 10.1107/S1600577515005317

**Published:** 2015-04-21

**Authors:** Gabriel Blaj, Pietro Caragiulo, Gabriella Carini, Sebastian Carron, Angelo Dragone, Dietrich Freytag, Gunther Haller, Philip Hart, Jasmine Hasi, Ryan Herbst, Sven Herrmann, Chris Kenney, Bojan Markovic, Kurtis Nishimura, Shawn Osier, Jack Pines, Benjamin Reese, Julie Segal, Astrid Tomada, Matt Weaver

**Affiliations:** a SLAC National Accelerator Laboratory, 2575 Sand Hill Road, Menlo Park, CA 94025, USA

**Keywords:** FEL, X-ray, detectors, fast readout

## Abstract

Free-electron lasers (FELs) present new challenges for camera development compared with conventional light sources. At SLAC a variety of technologies are being used to match the demands of the Linac Coherent Light Source (LCLS) and to support a wide range of scientific applications. In this paper an overview of X-ray detector design requirements at FELs is presented and the various cameras in use at SLAC are described for the benefit of users planning experiments or analysts looking at data. Features and operation of the CSPAD camera, which is currently deployed at LCLS, are discussed, and the ePix family, a new generation of cameras under development at SLAC, is introduced.

## Abstract 2

This paper offers an overview of area detectors developed for use at the Linac Coherent Light Source (LCLS) with particular emphasis on their impact on science. The experimental needs leading to the development of second-generation cameras for LCLS are discussed and the new detector prototypes are presented.

## 1. Introduction   

Along with the unprecedented new science opportunities at the Linac Coherent Light Source (LCLS) have come new challenges for instrumentation. Optics, diagnostics, detectors and data acquisition had to be developed to meet the new requirements. Many concepts established at storage rings cannot provide suitable solutions for the needs of this pulsed source. In particular, the extreme brightness, short pulses and 120 Hz repetition rate require detectors with fast readout and pose significant challenges for large two-dimensional cameras. Similarly to what is seen in other facilities, to fully exploit the source capabilities a corresponding effort in detector development was started (Koerner *et al.*, 2009 ▶; Carini *et al.*, 2009 ▶; Kameshima *et al.*, 2014 ▶; Hatsui, 2014 ▶; Henrich *et al.*, 2011 ▶; Porro *et al.*, 2010 ▶; Koch *et al.*, 2013 ▶; Mozzanica *et al.*, 2011 ▶, 2014 ▶).

As photons arrive all at once, the detector has to provide the intensity of each signal at a given position per pulse as opposed to counting the number of hits at a given position in a given period of time. X-ray CCDs provide intensity information with very good energy resolution. Although they are very common at synchrotrons, few CCD cameras can be read out at the speed required by the LCLS repetition rate. Two direct-conversion, back-illuminated, fully depleted X-ray CCD cameras with at least 120 Hz frame readout speed have been used at LCLS: the pnCCD, based on a pn-junction CCD sensor developed at the Halbleiterlabor (HLL, Germany), and the fCCD, based on a MOS CCD sensor developed at Lawrence Berkeley National Laboratory (LBNL) (Strüder *et al.*, 2010 ▶; Doering *et al.*, 2011 ▶). Thanks to their very low noise and good quantum efficiency over a large range of energies, they have been used in different operation modes for imaging and spectroscopy, providing single-photon and sub-pixel spatial resolution, in sparse data, in the soft and hard X-ray range (Chapman *et al.*, 2011 ▶; Gomez *et al.*, 2014 ▶; Loh *et al.*, 2012 ▶; Seibert *et al.*, 2011 ▶; Lee *et al.*, 2012 ▶; Chuang *et al.*, 2013 ▶; Johnson *et al.*, 2012 ▶; Först *et al.*, 2011 ▶). However, a larger maximum signal than these CCDs currently cover (a few hundred thousands electrons) is desired for many coherent imaging experiments.

The first detector specifically developed for use at LCLS is the Cornell–SLAC Pixel Array Detector (CSPAD) (Koerner *et al.*, 2009 ▶; Philipp *et al.*, 2010 ▶, 2011*a* ▶,*b* ▶; Hart *et al.*, 2012*a* ▶,*b* ▶). This detector is the result of a collaboration between Cornell University and SLAC National Accelerator Laboratory. Over the first five years of operation the CSPAD camera has reached maturity and has been the workhorse at the four LCLS hard X-ray hutches.

Lower noise and higher dynamic range cameras are needed to take full advantage of the science opportunities at LCLS. A new generation of cameras, the ePix, is being developed at SLAC for that purpose. These detectors also provide a path to fulfilling the needs of the upcoming LCLS-II (White *et al.*, 2015 ▶).

In the following we will discuss the use and application of the CSPAD at LCLS, then present the first two members of the ePix family, ePix100 and ePix10k (Blaj *et al.*, 2014*a* ▶).

## 2. CSPAD: the Cornell–SLAC Pixel Array Detector   

**Figure 1:**
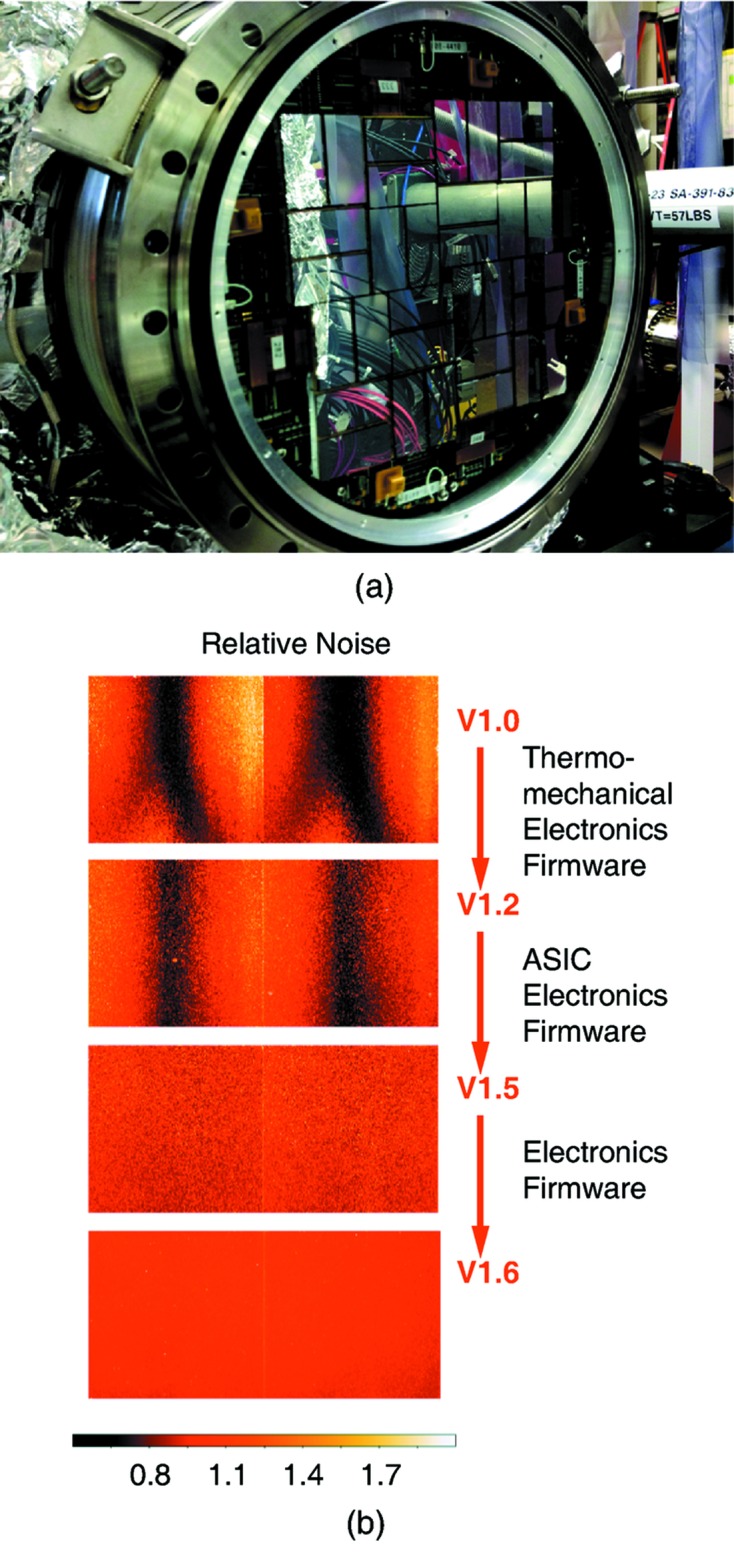
(*a*) CSPAD 2.3 Mpixel camera at the CXI instrument. (*b*) Relative normalized (unitless) noise per tile for the four different versions from V1.0 to V1.6.

The CSPAD is an integrating hybrid pixel array detector with a readout speed matched to the LCLS repetition rate (*i.e.*, 120 Hz). The main characteristics of the detector are reported in Table 1 ▶. The cameras are built around a module (or tile) comprising two ASICs bump-bonded to a pixelated silicon sensor. Over the past few years a series of upgrades have been implemented resulting in an improved and now mature detector (Herrmann *et al.*, 2012 ▶, 2013 ▶, 2014*a* ▶). The tiled structure of the camera and the relative noise improvement per tile are shown in Fig. 1 ▶.

**Figure 2:**
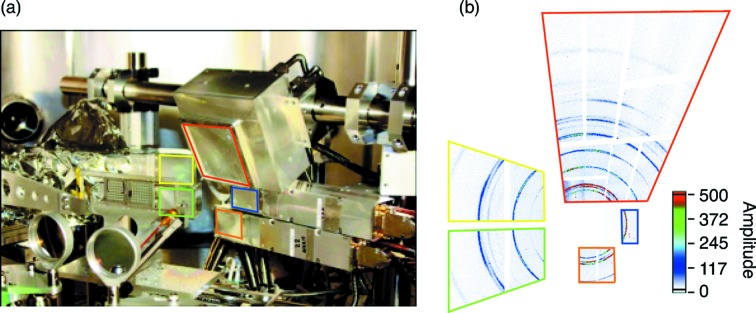
(*a*) MEC chamber: experimental setup with a CSPAD-560k and four CSPAD-140k. (*b*) Reconstructed diffraction rings from Ti samples at 10.2 keV in the various CSPAD panels. The red line indicates the location of the CSPAD-560k; and all other colors indicate the four distinct CSPAD-140k. The amplitude is in ADU. Courtesy of Cindy Bolme (Los Alamos National Laboratory).

We observed that the proximity of the X-ray beam, the sorts of samples studied and the requisite injection setups result in a high-damage environment for cameras (*e.g.* accidental exposure to focused or insufficiently attenuated beam, electromagnetic pulses generated by high-power optical lasers, *etc*.) (Blaj *et al.*, 2014*b* ▶), making highly modular design essential for quick repairs. This design paradigm is also important for high-yield production of large camera arrays. Large (∼17 cm × 17 cm), medium (∼8 cm × 8 cm) and small (5.8 cm × 4.7 cm × 21 cm, either 90° or front-facing) form-factor CSPAD cameras, with 2.3 M, 560k and 140k pixels, respectively, are available to satisfy the experimental needs. The cameras run at room temperature or slightly cooled, through the implementation of a short electronic shutter and power pulsing, simplifying deployment. Considering the increasing complexity of the experimental setups this is particularly important. The cameras operate in vacuum or air. An example is shown in Fig. 2 ▶ where a CSPAD-560k and four CSPAD-140k were simultaneously used in the Matter in Extreme Conditions (MEC) instrument to cover a large solid angle around the samples (Nagler *et al.*, 2015 ▶).

**Figure 3:**
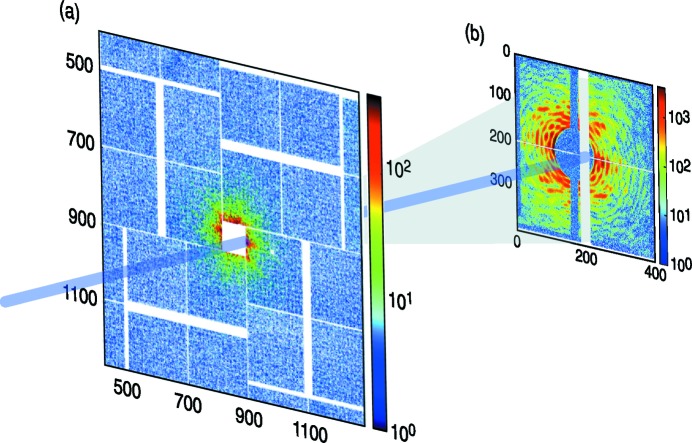
Single-shot coherent diffraction pattern of a single virus collected using 5.5 keV X-rays at the CXI instrument (Liang *et al.*, 2015 ▶). The color code shows the intensity in ADU. *X* and *Y* axes define the pixel positions. (*a*) Central part of a 2.3 Mpixel CSPAD use as the front detector to collect wide-angle scattering. (*b*) CSPAD-140K used as the back detector to measure diffraction that passes through the hole in the front. A beamstop was used to block the direct beam. Background subtraction was performed by Anton Barty from CFEL. Data were collected during the LC97 experiment led by Daniel Larsson (Uppsala University) using an aerosol injector to deliver samples to the beam.

Another typical application is shown in Fig. 3 ▶. In this case the large-area front detector is used to collect the wide-angle scattering while a second smaller detector, distant from the previous one, collects the small-angle scattering passing through the size-adjustable hole of the first camera. Accurate metrology, provided by the LCLS offline software, is essential to reconstruct the actual position of the signal in the camera. One of the main challenges of these experiments is the large variation of the measured intensities over a large *Q* range, which requires detectors with a wide dynamic range (Sellberg *et al.*, 2014 ▶). In the case of CSPAD this can be achieved by combining two fixed gain settings: the high gain provides low noise as needed for small signal detection, and the low gain allows a large maximum signal. The camera provides an effective electronic resolution of 11.4 bit and 12.6 bit in the high- and low-gain mode, respectively. As every single pixel in CSPAD can be programmed to either high- or low-gain mode we can utilize mixed gain configurations to optimize the camera for certain (static) illumination conditions and achieve in those cases a combined dynamic range and image fidelity of 14.4 bit. This value is limited by the Poisson process and possible crosstalk errors (Herrmann *et al.*, 2012 ▶).

**Figure 4:**
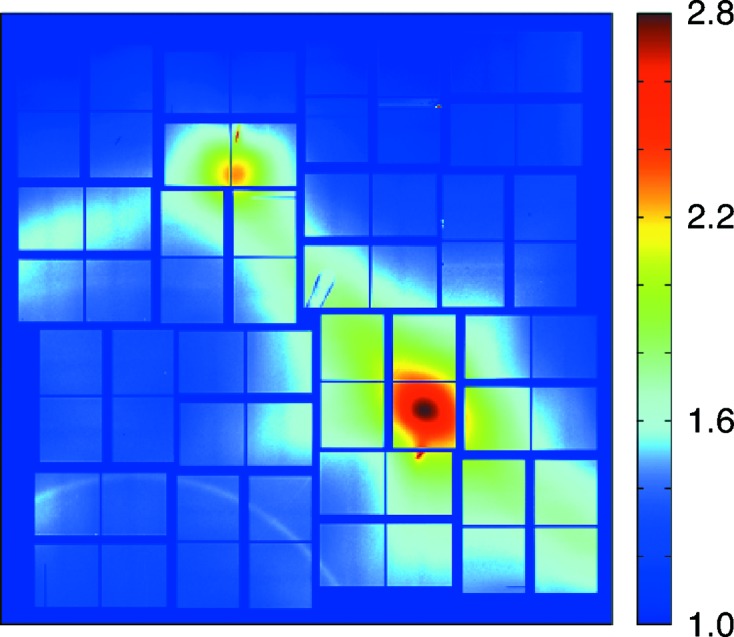
Static thermal diffuse scattering due to phonons in Ge. The image, in log10 scale, is an average of ∼1000 events, re-binned down to 512 × 512. Courtesy of Mariano Trigo (SLAC).

Pump–probe is another category of experiments very sensitive to amplitude errors (Trigo *et al.*, 2013 ▶). In this case the X-ray signal after excitation with an ultrashort laser pulse changes by much less than 1%. To overcome the Poisson fluctuation per shot at the CSPAD maximum signal, more than 2%, long data sets are collected, corrected (and re-binned) and averaged with the goal to reach better than the camera-noise-limited image quality. The noise is not fully random and uncorrelated. Measurements have shown that the camera noise does not behave as pure stationary random noise. The physical origin of other noise sources is still under investigation but pedestal and gain drift and crosstalk are major components limiting noise convergence; therefore only limited improvement can be expected (+2–3 bit) by averaging. An example of pump–probe data collected with the CSPAD is shown in Fig. 4 ▶.

As with essentially any detector, several corrections are applied to the CSPAD raw data. First, the level measured in the dark (‘dark image’) is subtracted from each pixel, and any pixels deemed too noisy, typically only a few per thousand at most, are masked. Then, when needed, a gain correction is applied. For the current version of the CSPAD camera the gain varies by about 5% for illuminations levels of a few 8 keV photons per pixel and becomes flat beyond ∼10 photons per pixel. Then a ‘common mode’ correction is applied to remove low-frequency noise in the readout electronics, *i.e.* frame-by-frame pedestal shift. For the CSPAD, where the correction is typically less than one-fifth of an 8 keV photon r.m.s. in the operating range, this is done on a per-ASIC basis; for the other cameras described in this paper (*i.e.* pnCCD and ePix) it is done on a per-row basis taking into account the bank structure of the readout chip and the illumination level. In the former case an algorithm tries to find a peak in the low end of the signal distribution and corrects the overall ASIC response. This technique is not effective with diffuse illumination, in which case one can apply a correction using a set of 22 unbonded pixels that only sample the frame common mode noise and not the X-ray signal. The common mode correction also acts as a first-order crosstalk correction. These corrections are available in the LCLS online and offline software. Additional crosstalk corrections can be applied (van Driel *et al.*, 2015 ▶).

One of the effects observed during the first years of operation of the CSPAD at LCLS was an incomplete charge collection in the case of a very intense signal. Since at an FEL all charges from the sensor arrive essentially simultaneously, the input stage of the readout electronics must be able to handle an abrupt voltage spike. Enough time (40 µs) should be given to the detector to respond properly, fully collecting the signal without losing charge. The latest version of the CSPAD camera with optimized tuning (*i.e.* V1.6) is much less susceptible to these effects (Carini *et al.*, 2014*b* ▶).

Today the CSPAD is the principal detector used for the LCLS hard X-ray experiments with more than 5 petabytes of data collected up to date.

## 3. Second-generation detectors for LCLS: the ePix family   

The general-purpose CSPAD cameras have worked well in addressing most of the hard X-ray needs of LCLS but some experiments need more specialized detectors. Potential future developments of the source into the tender X-ray regime, below 5 keV, and higher repetition rate, up to 100 kHz, will require new faster detectors. We are developing a family of cameras named ePix to meet these requirements (Blaj *et al.*, 2014*a* ▶).

These integrating hybrid pixel detectors fully exploit the modular design paradigm mentioned previously. The system and its components (ASICs, camera head, readout electronics and DAQ) have been designed to share SLAC standard interfaces, allowing quick development of the new cameras and corresponding online and offline analysis code. Different detectors, with characteristics matching the demands of specific classes of experiments, can be developed simultaneously and reuse many common parts such as electronic boards, firmware and software (Herrmann *et al.*, 2014*b* ▶). Scalability and hybrid combination of the single components are also natural advantages of this approach.

Also based on a common platform (*e.g.* reuse and sharing of design blocks, backend interface, *etc*.) is the ePix class of ASICs (Dragone *et al.*, 2013 ▶). This family of integrating architectures is composed of an analog matrix of pixels with global shutter and fast parallel column readout (100 kHz line rate) with multiplexed analog outputs. It also implements a dedicated control interface and all the required support electronics to perform configuration, calibration and readout of the matrix. The current series is designed to match the LCLS repetition rate but supports region of interest (ROI) mode, *i.e.*, the readout of a sub-set of the pixel array, for kHz frame rates. Versions with dedicated sigma–delta analog-to-digital converter per column are planned for future higher-repetition-rate experiments (kHz).

**Figure 5:**
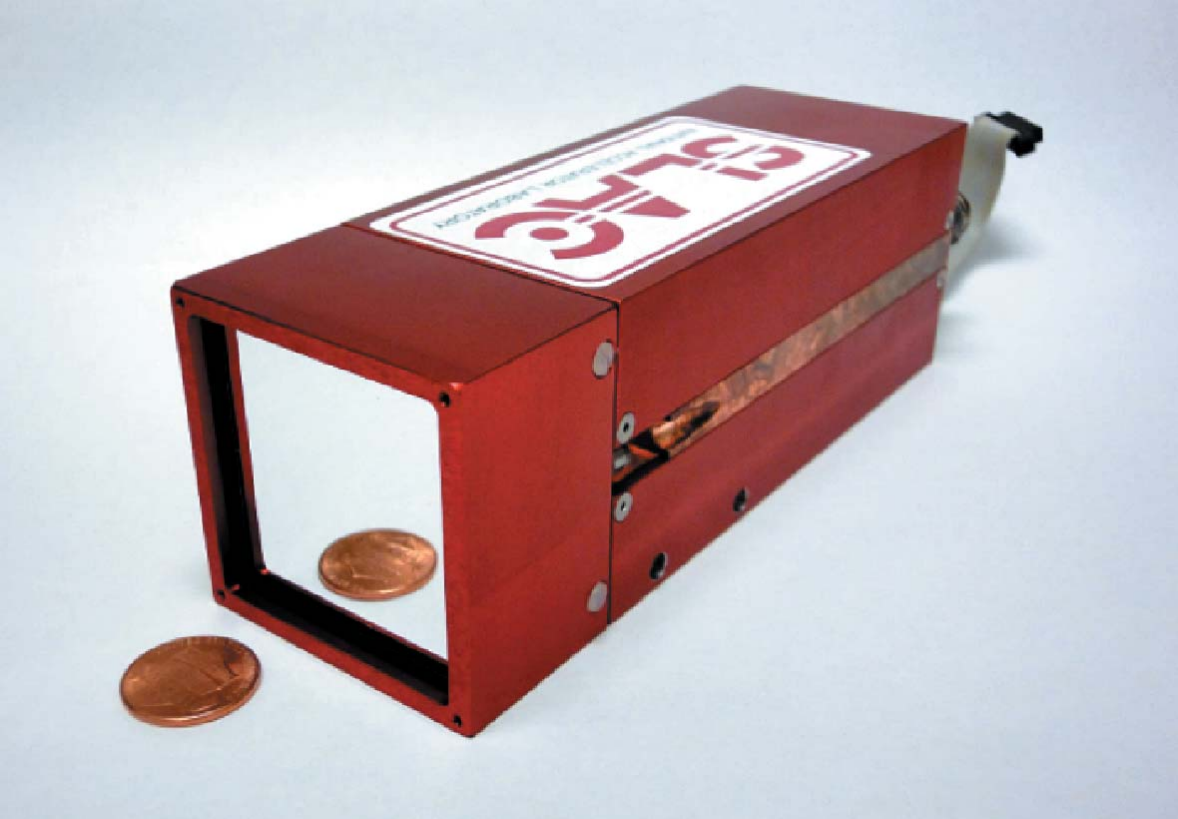
ePix camera. This assembly is used for both ePix100 and ePix10k detectors. It mounts a single silicon sensor with four bump-bonded ASICs, providing ∼0.5M and 130k pixels for each detector, respectively. This version of the camera is named ePix-ONE.

The first shared camera design is a very compact module of ∼52 mm × 52 mm × 155 mm (Fig. 5 ▶) with only 8 mm between the camera edge and the active area of ∼35 mm × 38 mm.

The camera head is modular and can be exchanged independently of the supporting analog and digital boards. The camera uses Peltier thermoelectric modules and water cooling in combination with dry nitrogen purge to prevent condensation. The data and control interface is realised *via* a fiber optical link. The software for online monitoring and visualization includes several important features such as for instance charge split reconstruction algorithms. Many experimental setups, such as crystal spectrometers in Rowland geometry and concurrent techniques using multiple detectors, are space constrained and benefit from a small compact camera, which can be placed easily with minimal impact on the rest of the experimental configuration. Greater radiation hardness and electromagnetic pulse (EMP) tolerance is expected thanks to the application of some radiation hardness techniques in the chip design (*e.g.* enclosed layout) and improved shielding for the camera housing.

### 3.1. ePix100   

The first member of this family is ePix100. The detector is designed to have low noise (better than 360 eV r.m.s.) and small pixels (50 µm × 50 µm) making it particularly suitable for X-ray photon correlation spectroscopy (XPCS) (Grübel *et al.*, 2008 ▶) and in combination with crystal spectrometers. As such it provides performance close to those of other direct-conversion scientific X-ray CCDs in use at LCLS combined with the typical advantages of hybrid pixel detectors (*i.e.* high frame-rate thanks to the pixel parallel amplification; room-temperature operation thanks to electronic shutter capability with short integration times at FELs; scalability, *etc*.). The name of the detector is representative of the maximum signal that the front-end can handle before saturation: this is the equivalent charge generated in silicon by one hundred 8 keV photons (∼35 fC). Noise considerations have driven the design and the optimization of the signal chain from the front-end onwards. Each pixel includes a single-stage low-noise charge integrator with a pulsed reset, a first-order non-linear programmable low-pass (Lp) filter, a correlated double sampler (CDS) and a sample-and-hold stage followed by a column buffer (Caragiulo *et al.*, 2014 ▶). Performance and detector characteristics are reported in Table 2 ▶.

The ePix100 prototype camera has been tested at the Stanford Synchrotron Radiation Lightsource (SSRL) and at LCLS. The prototype includes all the features of the full-size detector and mounts up to four ASIC-sensor modules. Sensor design and fabrication and bump bonding were carried out at SLAC and at the Stanford Nanofabrication Facility (SNF). Each bump-bonded prototype chip has 96 × 96 pixels.

**Figure 6:**
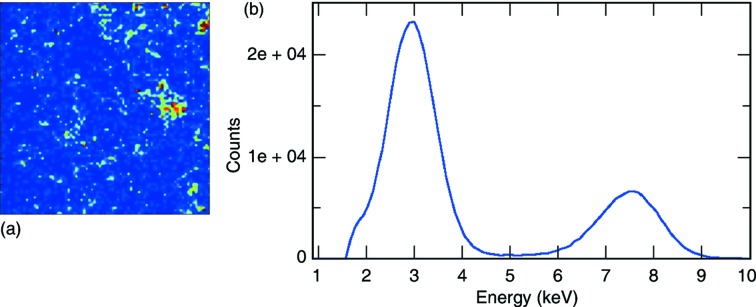
(*a*) Speckle pattern produced by 150 nm silica particles at the XCS instrument. The color code shows intensity in ADU. *X* and *Y* axes define pixels position. The data were collected with the first ePix100 prototype camera at ∼7.5 m from the sample using 8.54 keV X-rays. Courtesy of Marcin Sikorski (LCLS, SLAC). (*b*) Spectrum showing the Ag-*L* line and the primary energy (7.5 keV). Data collected at SSRL with the first ePix100 prototype.

In Fig. 6(*a*) ▶ a speckle pattern recorded with the prototype camera at the XCS instrument is shown (Alonso-Mori *et al.*, 2015 ▶). This is one of the scientific applications that really benefits from the detector characteristics. Its low-noise performance with single-photon signal-to-noise ratio of more than 25 at 8 keV makes the detector very efficient even in cases where the average photon rate is extremely low (*e.g.* smaller than 0.01 photons per 0.5 Mpixel frame), such as for instance XPCS at large wavector (*i.e.*, atomic resolution), and allows sub-pixel resolution to be achieved by interpolating the photon position from the measure charge cloud shared between four pixels, *e.g.* applying center of gravity algorithms. As a demonstration of the low-noise performance of the camera, Fig. 6(*b*) ▶ shows the Ag-*L* fluorescence line very well separated from the noise peak (Carini *et al.*, 2014*a* ▶). This spectrum is achieved from single pixel events with a threshold of 1.5 keV.

The linearity of ePix100 was measured both at SSRL and LCLS (XPP) and it is better than 0.2%. Ongoing calibration effort will allow further improvement on linearity. Poisson-limited images with 10% precision in a single shot at the maximum value of one-hundred 8 keV photons can be achieved. Recently the functionalities of a 384 × 352 pixel module have been tested and characterization with X-rays is ongoing.

The first version of the ePix camera with ePix100 modules will comprise four ASICs bump-bonded to a single silicon sensor (therefore called ePix-ONE) for a total of ∼0.5 Mpixel.

### 3.2. ePix10k   

**Figure 7:**
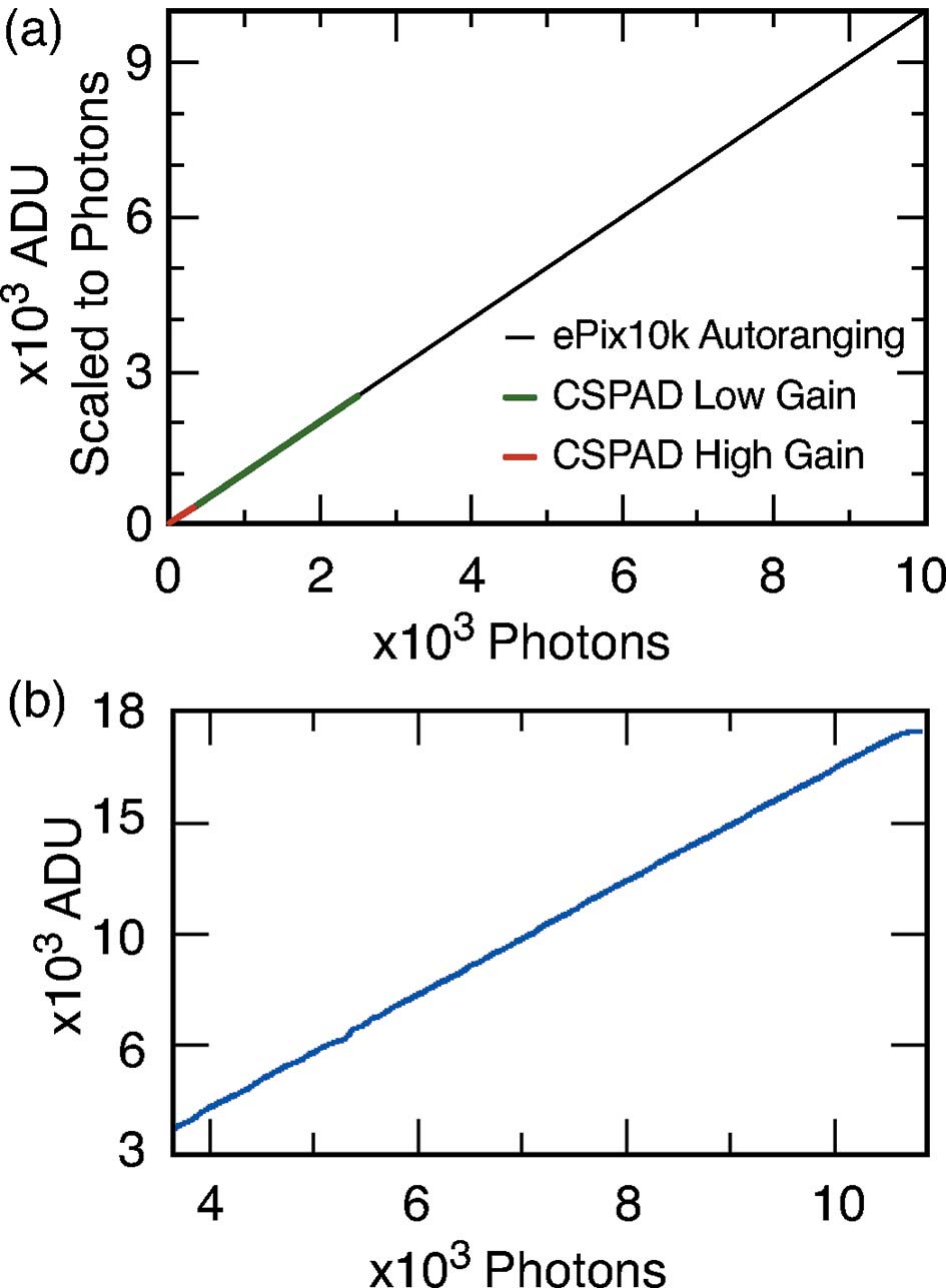
(*a*) The fixed high- and low-gain ranges of the CSPAD compared with the auto-ranging performance of the ePix10k (Monte Carlo simulation). (*b*) Correlation plot of the beam intensity measured with a beam monitor and the corresponding response of the ePix10k. Data were collected in low-gain mode with Cu *K* fluorescence.

The ePix10k is designed to replace the CSPAD for most hard X-ray imaging experiments, providing 10% better position resolution with at least one-third lower noise and a factor of four higher dynamic range [*cf*. Fig. 7(*a*) ▶] (Hart *et al.*, 2014 ▶). The ePix10k features a 100 µm × 100 µm pixel, also with single-stage low-noise charge integrator with a pulsed reset, a first-order non-linear programmable Lp filter, a CDS and a sample-and-hold stage followed by a column buffer. To provide a large dynamic range, the charge integrator makes use of an auto-ranging feature implemented with an additional 100× larger feedback capacitor and a comparator, similar to other architectures (Freytag *et al.*, 2008 ▶; Henrich *et al.*, 2011 ▶; Mozzanica *et al.*, 2014 ▶). When the comparator detects that the amplifier is approaching saturation, it switches in the additional capacitance, reducing the pixel gain by a factor of 100. Thus signals as large as ten thousand 8 keV photons can be acquired while still providing the capability to resolve single photons at low intensities. A 48 × 48 pixel module was fabricated in-house following the same steps as described above for the ePix100. Taking advantage of the family paradigm, the ePix10k module has been integrated into the ePix camera assembly and tested at SSRL and at the LCLS XPP instrument (Chollet *et al.*, 2015 ▶). A correlation plot of the beam intensity measured with a beam monitor and the the corresponding response of the ePix10k in low-gain mode is shown in Fig. 7(*b*) ▶; performance and detector characteristics are reported in Table 3 ▶.

Tests with very intense beam were also performed and confirmed the good stability of the detector under a large range of settings (Hart *et al.*, 2014 ▶). Analysis is ongoing and further measurements are being performed.

Thanks to the increased dynamic range, cameras based on ePix10k will extend the science reach of experiments like serial femtosecond crystallography and time-resolved pump–probe (Boutet *et al.*, 2012 ▶; Trigo *et al.*, 2013 ▶).

## 4. Conclusions   

The LCLS detector program has been part of the success of LCLS. CCDs are in use for imaging applications at the soft X-ray instruments. We have developed the CSPAD for a variety of imaging and spectroscopic applications in the hard X-ray hutches.

To make fuller use of the FEL capabilities we are developing the ePix family. The ePix10k will replace CSPAD in hard X-ray imaging experiments providing better position resolution, lower noise and higher dynamic range. The ePix100 will fulfill XPCS and spectroscopy experiments’ needs in the tender and hard X-ray range. It will provide noise performance and spatial resolution close to those of fast direct-conversion X-ray CCD cameras with the additional advantages of a hybrid pixel array detector, particularly scalability and simpler operation and deployment.

## Figures and Tables

**Table 1:** CSPAD characteristics and measured performance

CSPAD	High gain		Low gain
Pixel per ASIC		194 185	
Pixel size (m)		110	
Noise r.m.s. (eV)	1000		3500
Maximum signal (8keV photons equivalent)	350		2700
Frame rate (Hz)		120	
Sensor thickness (m)		500	

**Table 2:** ePix100 characteristics and measured performance

Pixel per ASIC	384 352
Pixel size (m)	50
Noise r.m.s. (eV)	360
Maximum signal (8keV photons equivalent)	100
Frame rate (Hz)	120
Sensor thickness (m)	300/500

**Table 3:** ePix10k characteristics and performance measured on the 48 48 pixel prototype

	High gain		Low gain
Pixel per ASIC		192 176	
Pixel size (m)		100	
Noise r.m.s. (eV)	650		10800
Maximum signal (8keV photons equivalent)	100		10000
Frame rate (Hz)		120	
Sensor thickness (m)		500	
